# When Anti-Neutrophil Cytoplasmic Antibody Fails: A Case of Anti-Neutrophil Cytoplasmic Antibody Negative Granulomatosis With Polyangiitis

**DOI:** 10.7759/cureus.8883

**Published:** 2020-06-28

**Authors:** Mounika Gangireddy, Tejaswi Kanderi, Janet Chan Gomez, Vishwa Kundoor, Jessica Cunningham

**Affiliations:** 1 Internal Medicine, University of Pittsburgh Medical Center (UPMC) Pinnacle, Harrisburg, USA; 2 Internal Medicine, MedStar Franklin Square Medical Center, Baltimore, USA

**Keywords:** anca negative, diagnostic criteria, renal biopsy, rituximab, granulomatosis with polyangiitis, glomerulonephritis

## Abstract

Granulomatosis with polyangiitis (GPA) is a vasculitis of small and medium-sized vessels and presents with varying signs and symptoms. It includes upper and lower airway manifestations and glomerulonephritis with a positive antineutrophil cytoplasmic antibody (ANCA) in serology in 90% of cases. However, about 10% of cases with GPA can have negative serology, often resulting in a diagnostic delay. Obtaining a tissue pathology is needed to confirm GPA. Here we present a 77-year-old male who presented with generalized weakness and loss of appetite and was found to have glomerulonephritis and bilateral opacities in the lungs with a negative ANCA. He was diagnosed with ANCA negative granulomatosis with polyangiitis after a renal biopsy revealed necrotizing inflammation with crescent formation. He was successfully treated with systemic glucocorticoids and rituximab. In conclusion, prompt diagnosis and treatment of ANCA negative vasculitis are required to decrease mortality.

## Introduction

Granulomatosis with polyangiitis (GPA) is a subtype of antineutrophil cytoplasmic antibody (ANCA)-associated vasculitis, which commonly affects small and medium-sized vessels. It results in an immune-mediated tissue injury driven by high titers of antibodies against human cytoplasmic granule proteins of neutrophils (ANCA) [1].

ANCA positivity has been correlated with clinical manifestations, risk of flares, and even treatment responsiveness in addition to being a diagnostic marker and is thought to be responsible for the pathogenesis of GPA until cases of ANCA negative GPA have been reported [2, 3]. Indirect immunofluorescence detects two types of ANCA, diffuse cytoplasmic (c-ANCA) and perinuclear/nuclear (p-ANCA).

The clinical manifestations of GPA can be diverse. It can involve upper and lower airway tracts, glomerulonephritis, skin, and blood vessels. The peak incidence occurs at the age of 64-75. However, there is a higher incidence of ANCA negative vasculitis in the younger population with an average of 54 years as reported in a retrospective study [4, 5]. Serological and histopathological confirmation is often needed for the diagnosis of GPA. Renal pathology is characteristic of the crescent formation along with necrotizing inflammation with no or few immune deposits. When other organs are involved, necrotizing granulomatous inflammation is noted.

Prompt diagnosis of GPA is important as the untreated disease is reported to have a fatal course with only 10% surviving at two years and mean survival of five months if untreated [6]. A cohort study done by Shah et al. demonstrated that 30% of ANCA positive patients had a diagnosis of GPA before renal biopsy whereas no ANCA negative patients were assigned a diagnosis. Given the mortality associated with the disease, being aware of ANCA negative disease can have a significant impact on early diagnosis and management [7].

Here we describe a 77-year-old male who presented with generalized weakness and was found to have glomerulonephritis and bilateral lung opacities and was ultimately diagnosed with ANCA negative GPA.

## Case presentation

A 77-year-old male with a medical history of chronic obstructive pulmonary disease (COPD), insulin-dependent diabetes type 2, hypertension, and benign prostatic hypertrophy presented to the emergency department complaining of generalized weakness for the past two months. Apart from generalized weakness, a review of systems was negative. Of note, he was treated with antibiotics for community-acquired pneumonia a month before the presentation.

Vital signs on admission included a temperature of 36.6 C, heart rate of 96 beats/minute, systolic blood pressure of 159/99 mm Hg, respiratory rate of 18/minute, saturating at 100% on room air. Physical examination revealed diminished bilateral breath sounds, normal S1, S2, no pedal edema, or focal neurological deficits. Labs are represented in Table 1 below.

**Table 1:** Representing lab values on admission.

Lab	Value	Reference
Hemoglobin	8.7 g/dl (baseline 10)	13-15 g/dl
WBC	16.50 k/uL	4-10 k/ul
Hematocrit	27.40%	38-49
Platelets	392 k/uL	150-350 k/ul
Bicarbonate	22.7 mmol/l	20-33 mmol/l
Anion gap	16.3 mg/dl	<12 mg/dl
BUN	51 mg/dl	7-25 mg/dl
Creatine	4.60 mg/dl	0.80 mg/dl
e-GFR	13 ml/min/1.73 sqm	70-90 ml/min/1.73 sqm
ESR	119 mm/hr	0-30 mm/hr
CRP	5.69 mg/dl	0-1 mg/dl
CK	42 u/l	30-150 u/l

Urinalysis (UA) revealed +3 blood (reference - negative), +2 protein (reference - negative), RBC > 50 (reference 0-5), urine ph of 6.0 (reference 5-8), fractional excretion of sodium (Fe-Na) is 1.7% (normal < 1%).

**Figure 1:**
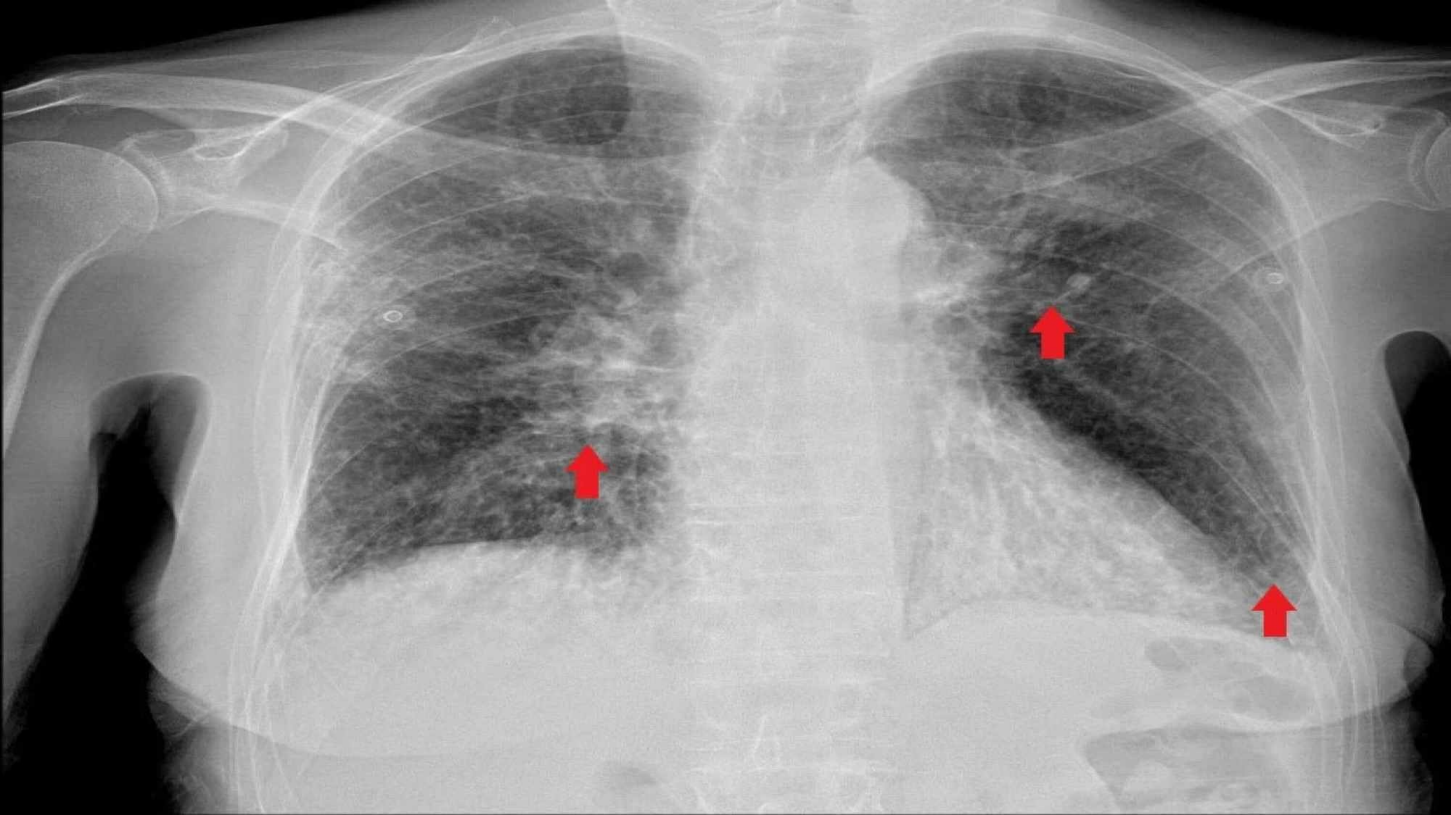
Chest X-ray showing multifocal infiltrates.

**Figure 2:**
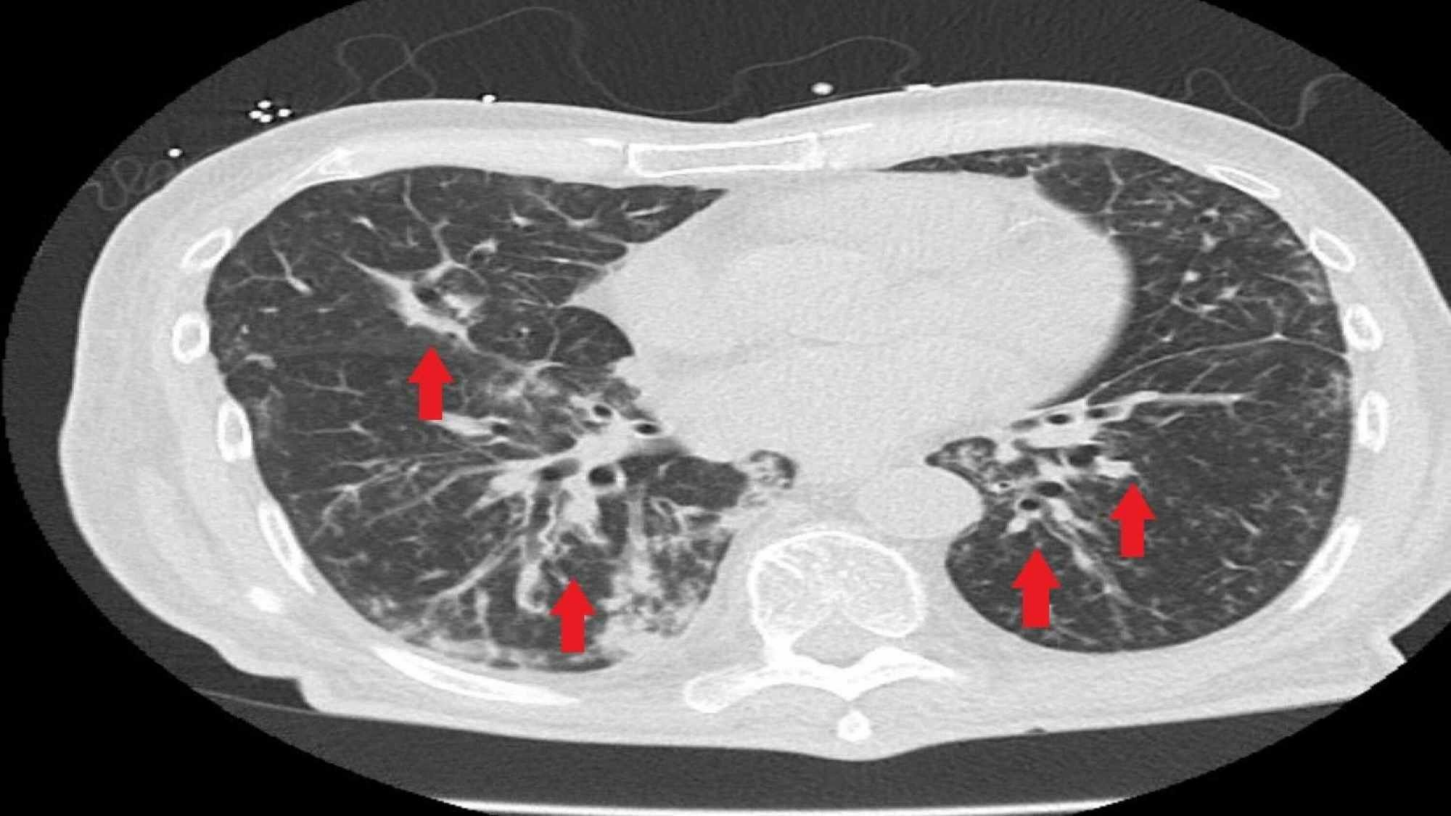
CT chest revealing multifocal densities and bronchiectasis.

The chest X-ray was consistent with multifocal infiltrates (Figure 1). CT chest without contrast demonstrated multifocal pulmonary densities compatible with areas of consolidation, greatest in the lower lobes along with bilateral bronchiectasis (Figure 2). Retroperitoneal ultrasound revealed no hydronephrosis or acute abnormalities. The bladder was decompressed with a foley catheter followed by a steady urine output of about 0.5 ml/kg/hour.

Differentials included acute glomerulonephritis, acute tubular nephritis, infiltrative disease, or vasculitis. On further review, he had multiple admissions in the past year for recurrent pneumonia requiring intravenous antibiotics every time. Serological workup for vasculitis revealed negative antinuclear antibody (ANA), negative anti-GBM antibody (glomerular basement membrane antibody), C3 104 mg/dl (reference 80-160), C4 17 mg/dl (reference 15-45), ELISA anti-Pr3 (Proteinase 3) <0.7 U (reference > 1.9), ELISA anti-MPO (Myeloperoxidase) <0.3 U (reference < 3.4). Bronchoscopy showed multiple areas of mucous plugging, and bronchial lavage with cultures negative for acid fast stain, bacterial, fungal, or viral growth. Kidney biopsy was performed given the high suspicion for ANCA-associated vasculitis.

**Figure 3:**
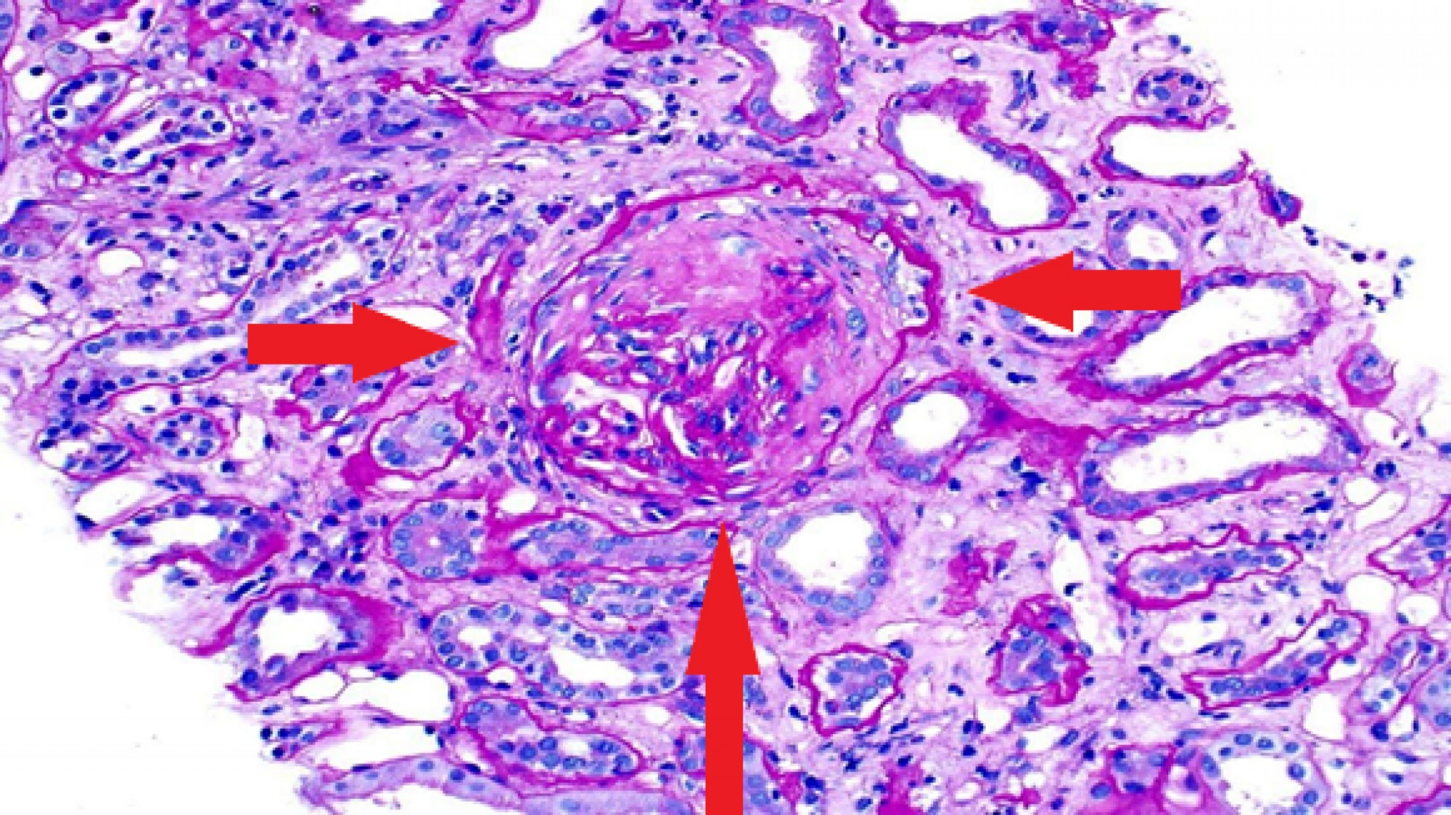
Renal biopsy illustrating fibrous crescent obliterating glomeruli.

**Figure 4:**
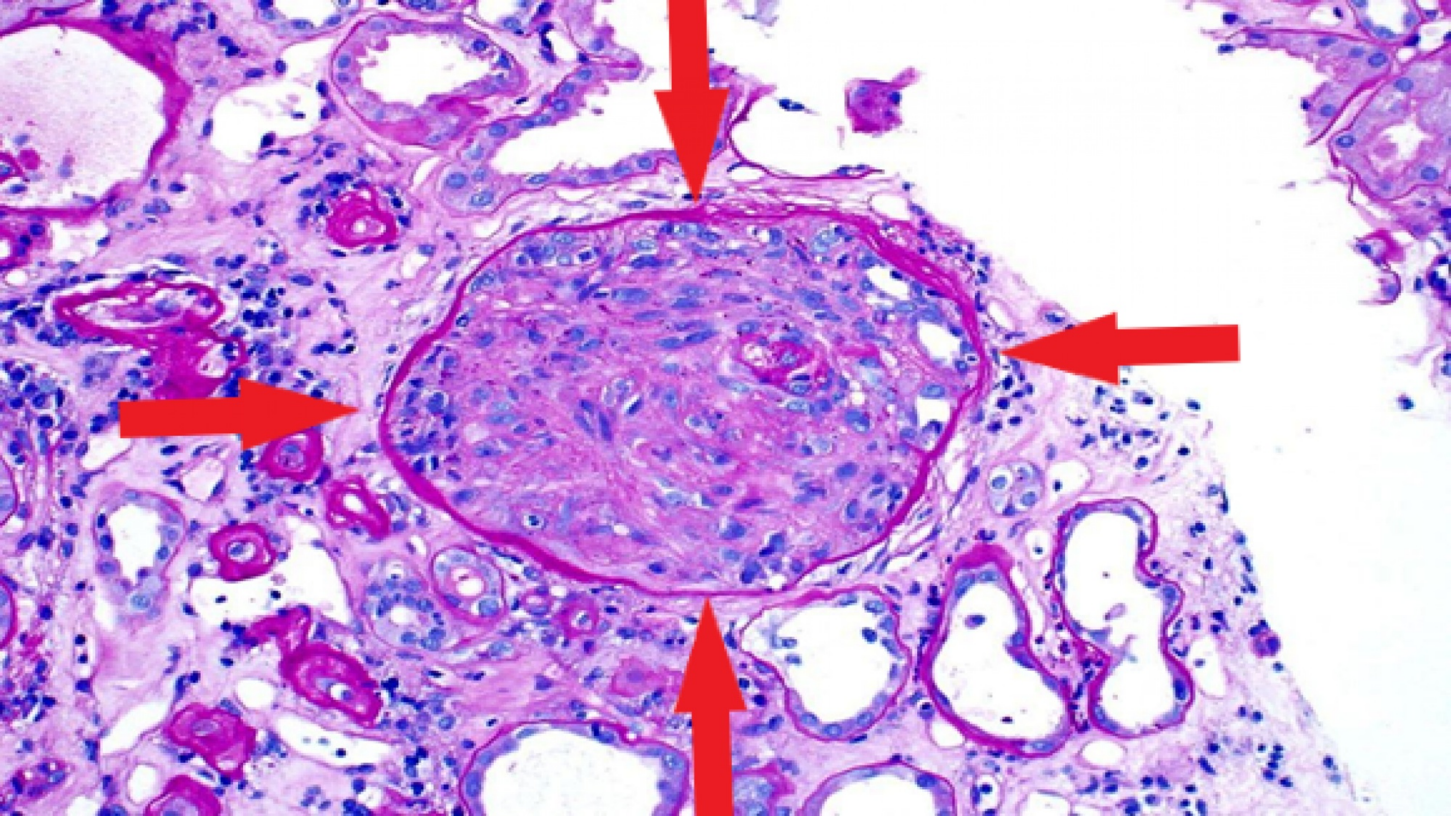
Renal biopsy representing the circumferential crescent.

In the meantime, he was started on pulse dose intravenous methylprednisolone 1 gm every 24 hours for three days followed by oral prednisone 60 mg daily. Tissue pathology revealed focal necrotizing and crescentic glomerulonephritis, pauci-immune type (Figures 3, 4). Based on the results of renal pathology, the patient was diagnosed with ANCA negative glomerulonephritis. The nephrology team initiated the monoclonal CD-20 inhibitor, rituximab, as the patient's renal function had not improved despite high dose steroids. The dose of rituximab was 375 mg/m^2^ weekly for four doses. He was discharged on steroid taper with nephrology follow-up outpatient.

Following the completion of treatment with rituximab, renal function stabilized with an eGFR of 25 ml/min/1.73 sqm (chronic kidney disease 4) and proteinuria decreased significantly. He is currently on surveillance.

## Discussion

We used the American College of Rheumatology (ACR) criteria and the European Medicine Agency (EMA) algorithm to classify GPA. Our patient was diagnosed with GPA given the findings on urinalysis and chest X-ray. If two out of the four criteria of ACR are positive, sensitivity is 88% and specificity is 92% [8, 9]. Given the fixed infiltrate on the chest X-ray for more than a month and the presence of glomerulonephritis, he was diagnosed with GPA per EMA algorithm as well.

GPA is usually positive for either PR3-ANCA, a type of c-ANCA (65%), or MPO, a type of p-ANCA [10]. ANCA is noted to be present in almost all patients with severe disease but only 80% of patients with limited disease have ANCA [11]. On the contrary, our patient's ANCA was negative despite renal and extrarenal manifestations.

Tissue diagnosis of active sites plays a crucial role in the confirmation of GPA. In most cases of ANCA positivity, a biopsy is not required for the initiation of treatment. However, if ANCA is negative, a biopsy is warranted to guide the diagnosis. Histological findings in ANCA negative disease are similar to that of ANCA positive disease [2]. Renal pathology in our patient was consistent with necrotizing vasculitis with crescent formation, confirming ANCA negative pauci-immune crescentic glomerulonephritis (CrGN).

Pauci-immune CrGN usually occurs as a complication of a GPA [2]. It occurs in association with ANCA positive vasculitis either PR3-ANCA or MPO ANCA. As described by some studies, ANCA positivity has a sensitivity and specificity of >90%, however 10-30% of cases with pauci-immune CrGN lack ANCA [1, 12]. In our patient, ANCA was negative but the biopsy was consistent with pauci-immune crescentic glomerulonephritis.

Retrospective analysis has reported that the etiology of ANCA negative CrGN is not entirely clear, but the pathogenesis is thought to be due to neutrophil infiltration. Although the idea of the existence of AECA and other autoantibodies with close characteristics of ANCA cannot be ruled out [1, 3, 13]. Literature also notes a relationship between inflammatory cytokines like interleukins (IL6) and ANCA negative vasculitis [14]. Zhou et al. found an association between lack of CCR5 deletion and ANCA negativity [15]. Further studies establishing the association between markers and ANCA negative vasculitis are needed to expand treatment options [16].

Management of GPA with organ involvement has been divided into induction and maintenance therapy. Induction therapy includes high dose glucocorticoids and immunotherapy with either rituximab or cyclophosphamide [12]. Maintenance therapy includes low-level immunosuppression with low dose glucocorticoids with or without rituximab. There are no studies comparing the treatment for ANCA positive and ANCA negative vasculitis [2, 17]. Therefore, the same treatment protocols are used in ANCA negative patients. Our patient was started on high dose glucocorticoids while awaiting biopsy.

Rituximab is a monoclonal antibody against CD 20 antibody found to be effective even in antibody (ANCA) negative disease as reported in the literature. It is likely due to the involvement of B cells in the inflammatory process by mechanisms like antigen presentation or antibody production other than ANCA owing to treatment responsiveness [18]. Further studies are needed to establish the mechanism of efficacy in antibody-negative disease.

In cases of ANCA positivity, titers are useful in monitoring disease activity and follow-up, however management is not solely based on the titers. Lack of monitoring in ANCA negative patients may result in delay or difficulty in identifying flares.

## Conclusions

Overall we feel it is important for clinicians to be aware of ANCA negative vasculitis to aid in prompt management. Diagnosis of ANCA negative GPA without biopsy is a challenge due to a lack of serum markers. However, using the American College of Rheumatology criteria and European Medicine Agency (EMA) algorithm, and having a high index of suspicion to consider ANCA negative vasculitis as a differential is important for early diagnosis and treatment to improve outcomes.
